# Overlapping RdDM and non-RdDM mechanisms work together to maintain somatic repression of a paramutagenic epiallele of maize *pericarp color1*

**DOI:** 10.1371/journal.pone.0187157

**Published:** 2017-11-07

**Authors:** Po-Hao Wang, Kameron T. Wittmeyer, Tzuu-fen Lee, Blake C. Meyers, Surinder Chopra

**Affiliations:** 1 Department of Plant Science, Pennsylvania State University, University Park, Pennsylvania, United States of America; 2 Plant Biology Program, Pennsylvania State University, University Park, Pennsylvania, United States of America; 3 Department of Plant & Soil Sciences and Delaware Biotechnology Institute, University of Delaware, Newark, Delaware, United States of America; CNRS UMR7622 & University Paris 6 Pierre-et-Marie-Curie, FRANCE

## Abstract

Allelic variation at the *Zea mays* (maize) *pericarp color1* (*p1*) gene has been attributed to epigenetic gene regulation. A *p1* distal enhancer, 5.2 kb upstream of the transcriptional start site, has demonstrated variation in DNA methylation in different *p1* alleles/epialleles. In addition, DNA methylation of sequences within the 3’ end of intron 2 also plays a role in tissue-specific expression of *p1* alleles. We show here a direct evidence for small RNAs’ involvement in regulating *p1* that has not been demonstrated previously. The role of *mediator of paramutation1* (*mop1*) was tested in the maintenance of somatic silencing at distinct *p1* alleles: the non-paramutagenic *P1-wr* allele and paramutagenic *P1-rr*’ epiallele. The *mop1-1* mutation gradually relieves the silenced phenotype after multiple generations of exposure; *P1-wr*;*mop1-1* plants display a loss of 24-nt small RNAs and DNA methylation in the 3’ end of the intron 2, a region close to a *Stowaway* transposon. In addition, a MULE sequence within the proximal promoter of *P1-wr* shows depletion of 24nt siRNAs in *mop1-1* plants. Release of silencing was not correlated with small RNAs at the distal enhancer region of the *P1-wr* allele. We found that the somatic silencing of the paramutagenic *P1-rr*’ is correlated with significantly reduced H3K9me2 in the distal enhancer of *P1-rr*’; *mop1-1* plants, while symmetric DNA methylation is not significantly different. This study highlights that the epigenetic regulation of *p1* alleles is controlled both via RdDM as well as non-RdDM mechanisms.

## Introduction

Epigenetic gene regulation affects genome integrity by maintaining, over generations, silencing in repetitive sequences and transposons [1]. In plants, epigenetic regulation plays a key role in phenomena such as genomic imprinting [2] and paramutation [3–7] mainly, by enforcing a silenced chromatin state through DNA methylation and/or chromatin modifications, leading to gene silencing. The silenced states are then maintained transgenerationally by epigenetic machinery [8]. Several components involved in the maintenance of transcriptional gene silencing have been identified in plants, the detailed mechanisms that may be distinct among different plant species are not fully understood.

Paramutation has remained an intriguing epigenetic process; it is defined as an interaction between two alleles, in which a silenced (somatic repression) allele suppresses a homologous active allele in *trans* (establishment) and leads to a heritable change in expression [6,9]. The newly repressed allele is meiotically heritable and is capable of suppressing another homologous naïve allele. Paramutation was first observed in the maize *r1* (*red1*) gene [10], and later found in *b1* (*booster1*) [11], *pl1* (*purple plant1*) [12], *p1* (*pericarp color1*) [13], and *lpa1-241* (*low phytic acid1*) [14]. Paramutation-like phenomena have also been reported in other plants, fungi, and animals (For review see [9]). The mechanism of paramutation in maize has been extensively characterized at the *b1* locus, but many details remain elusive. Seven direct tandem repeats located 100 kb upstream of the *b1* coding region are critical and sufficient to mediate paramutation [15,16]. These tandem repeats demonstrate differential DNA methylation and chromatin states between paramutable (*B-I*) and paramutagenic (*B’*) alleles [15,17]. Several genes involved in maize paramutation have been identified and these turned out to be homologs of components involved in RNA-directed DNA methylation (RdDM) in *Arabidopsis*. These genes are required for various aspects of paramutation and siRNA biogenesis, including *mediator of paramutation1* (*Mop1*) (encodes RDR2, an RNA-dependent RNA polymerase) [18], *required to maintain repression6* (*Rmr6)/Mop3* (encodes NRPD1, the largest Pol IV subunit) [19,20], *Mop2/Rmr7* (encodes NRPD2/E2, the second largest Pol IV and Pol V subunits) [21,22], *Rmr1* (encodes a Rad54-like ATPase) [23], and *rmr2* (encodes a novel plant specific protein) [24]. Mutations in these genes disrupt paramutation and drastically reduce the abundance of 24-nt heterochromatic siRNAs (“siRNAs” hereafter). This implies that a siRNA-mediated mechanism is involved in paramutation although siRNAs alone are not sufficient for paramutation in certain cases [16,25].

The maize *Mop1* gene is required for the establishment of paramutation at *r1*, *b1*, *pl1*, and *p1* loci [18,26]. However, *Mop1* differentially maintains somatic repression at these loci; the *mop1-1* mutation disrupts the silencing at *B’* and *Pl’*, but has a gradual effect at *P1-rr*’ [18,26]. Additionally, *Mop1* is involved in the maintenance of transcriptional silencing at *Mutator* (*Mu*) transposons, transgenes, and the non-paramutagenic *P1-wr* allele [18,27–29]. Notably, it takes multiple generations in the presence of *mop1-1* to disrupt the silencing of *MuDR* and *p1* alleles [18,30]. Another component encoded by *Mop2/Rmr7* (NRPD2/E2) also shows different requirements for establishment and somatic repression of various paramutagenic alleles. The *Mop2-1* mutation contained an amino acid change in a highly conserved motif essential for polymerase activity in the second largest subunit of Pol IV and Pol V [21]. Paramutation of *B-I* to *B’* is abolished by *Mop2-1* in a dominant fashion, while somatic repression is released at *B’* in a recessive manner. However, *Mop2-1* acts recessive for establishment of paramutation at *P1-rr*’ and does not release somatic repression of *P1-rr*’ even after three generations of exposure [21]. By contrast, maize *rmr1* (*required to maintain repression1*) encodes a SNF2 protein and is required for accumulation of a majority of 24-nt siRNAs, plays a role in maintaining the repressed chromatin states at *Pl’* and transgenes, but it is not required for establishment of the *Pl’* paramutation [23,28].

The above-mentioned genetic interactions exemplify the complexity of the mechanisms underlying the establishment of paramutation and maintenance of somatic repression, demonstrating that components of silencing machinery are not always shared between the two processes. In maize, another well-characterized gene system, *p1*, offers an opportunity to dissect the mechanism required for the establishment and maintenance of paramutation. The *p1* gene encodes an R2R3 MYB transcription factor which regulates the 3-deoxyflavonoid biosynthetic pathway leading to the accumulation of red phlobaphene pigment in floral tissues [31,32]. Multiple alleles of *p1* can be identified by their distinct expression patterns in pericarp and cob glumes. For instance, *P1-wr* specifies *w*hite pericarp and *r*ed cob and *P1-rr* conditions *r*ed pericarp and *r*ed cob tissues [33]. Paramutation at *P1-rr* has arisen independently twice; once spontaneously as *P1-pr* [34,35] and once through transgene induction of *P1-rr*’ [13]. The sequences required for paramutation were identified as being part of a transcriptional enhancer called the P1.2/distal enhancer (DE) [13,36]. The P1.2 contains a MULE fragment and *p1* repeats and is present ~5 kb upstream of the TSS and repeated twice at the 3’ end of the gene. Paramutation of *P1-rr* is not fully penetrant and can lead to a range of pericarp pigmentation phenotypes. The amount of reduction of pigmentation in *P1-rr*’ is inversely correlated with both increased paramutagenicity and increased DNA methylation of the DE [13,35].

The silent pericarp phenotype of *P1-wr* is extremely stable and correlates with hypermethylation of the distal enhancer (DE) region, which is located between 5135 and 4637 bp upstream of the TSS [37] and overlaps with the P1.2 promoter element of *P1-rr* identified as an enhancer in transgenic experiments [36]. In addition to *P1-wr*, other silenced *p1* epialleles such as *P1-pr* [38], *P1-rr*’ and *P1-pr*^*TP*^[39], *p1-ww*:*DP* [40], and P1-wr* [41] are epigenetically regulated. A wealth of information related to epigenetic regulation of *p1* has come from previous studies involving the dominant *Unstable factor for orange1* (*Ufo1*) mutation which is involved in maintaining somatic repression of silenced alleles. These studies of *P1*-*Ufo1-1* interactions have established different regions of *p1* associated with changes of DNA methylation and histone modifications in different *p1* alleles and epialleles. For example, hypomethylation of the DE correlates with increased transcription of *p1* in pericarp and thus leading to enhanced pigmentation and hypomethylation of sequences in the intron 2 region lead to a gain of cob pigmentation [38–43].

The current study was designed to understand the role of epigenetics in maintenance of allelic expression patterns observed in different alleles/epialleles of *p1* in maize. The specific questions addressed are: (1) Are there separate mechanisms for the regulation for *p1* alleles/epialleles that have been derived via paramutation or non-paramutation types of gene silencing mechanisms? (2) Are *p1* alleles regulated via the RdDM pathway? (3) Why do the requirements of somatic repression of *P1-rr*’ differ from other paramutagenic loci? To address these questions, changes in epigenetic states were compared at the paramutagenic *P1-rr*’ and non-paramutagenic *P1-wr* alleles in the presence or absence of *mop1-1*. This study provides evidence for the regulation of *p1* alleles by RdDM and chromatin modifications.

## Results

### *mop1-1* affects DNA methylation of an intron 2 region of *P1-wr*

**Fig 1:**
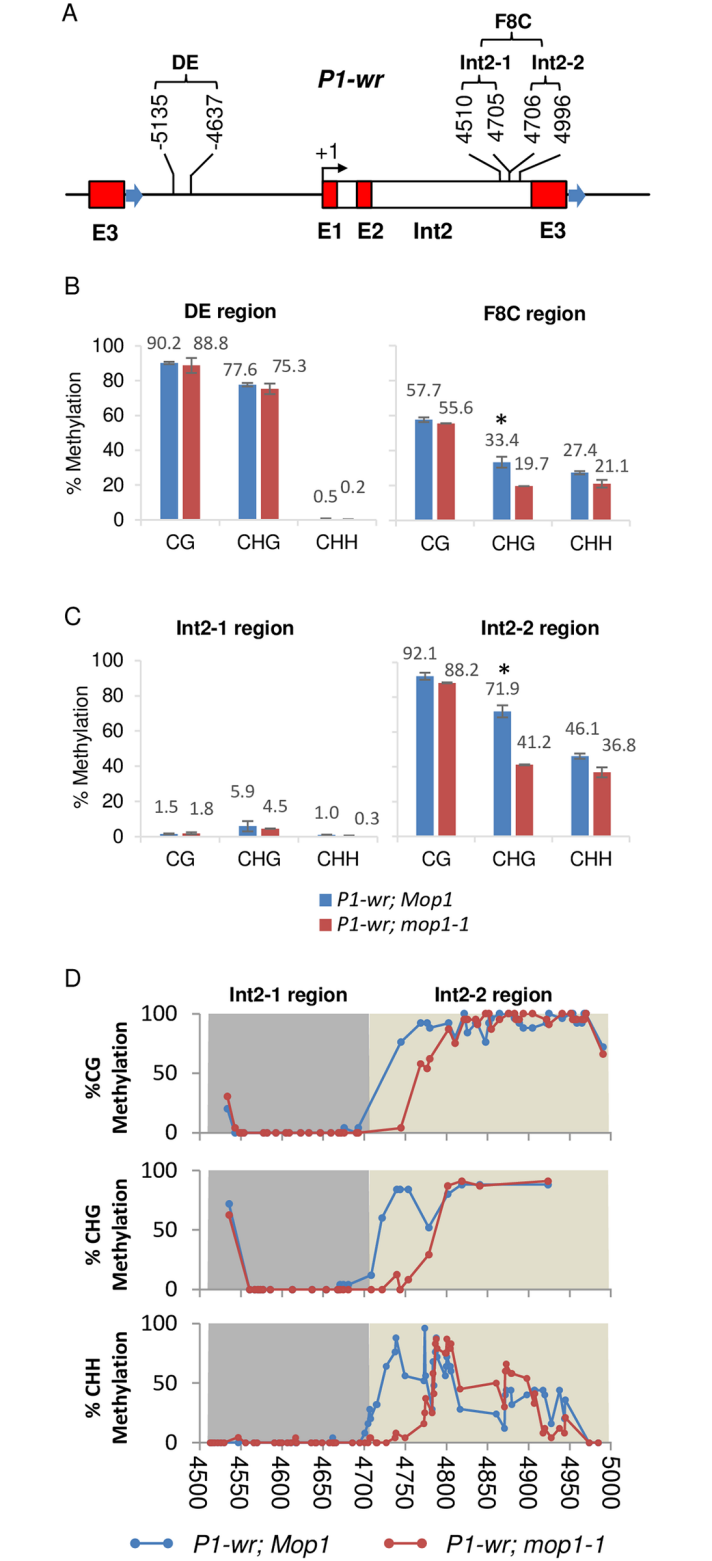
Cytosine methylation level of two regulatory regions of *P1-wr*. (A) Line diagram representing *P1-wr* gene structure. Block arrows represent sequences of tandem repeat.. The transcription start site is shown as a bent arrow marked +1. Red boxes labeled E1, E2 and E3 represent exon 1, 2 and 3, respectively. Introns are open boxes. Sequence coordinates of DE and F8C regions analyzed by bisulfite sequencing are shown above the gene structure (accession no. EF165349). (B) Overall DNA methylation level at CG, CHG and CHH (H is A, C, or T) contexts for DE and F8C regions in *mop1-1* (*P1-wr*/*P1-wr*; *mop1-1*/*mop1-1*) and *Mop1* (*P1-wr*/*P1-wr*; *Mop1*/*mop1-1*) plants. Average methylation is calculated as the mean of at least two biological replicates with error bars indicating the SE of the mean. * represents means are significantly different (*P* ≤ 0.05) as determined by Student’s *t* test. (C) Comparison of DNA methylation changes at *P1-wr* sequences within Int2-1 and Int2-2 regions of F8C in the presence of *mop1-1*. Percent methylation is shown on the y-axis. (D) DNA methylation of individual cytosines (circles) of the Int2-1 and Int2-2 regions.

The stably inherited *P1-wr* colorless pericarp phenotype can be altered in the presence of the *mop1* mutation after multiple generations of exposure [18]. The gain of the pericarp pigment phenotype observed in *P1-wr*; *mop1-1* was attributed to RNA-based mechanisms that may participate in tissue-specific silencing, however no molecular mechanism has been demonstrated for *P1-wr*; *mop1-1* reactivation. To determine the underlying cause of *mop1*-1-induced reactivation of *P1-wr* pericarp expression, we performed genomic bisulfite sequencing for DNA methylation analysis on two regulatory regions, the DE and the 3’ end of intron 2 (F8C) of *p1* (Fig 1). The DE and F8C regions are known to contain *cis*-acting transcriptional enhancer elements for *p1* expression in pericarp [36,43] and cob glume pigmentation [41], respectively. The epigenetic state of these regulatory regions can also be affected by *Ufo1-1*, which induces transcriptional reactivation associated with hypomethylation [41,43]. We found that the *P1-wr* DE region remained hypermethylated in *mop1-1* as compared to *Mop1* plants (Fig 1B and S1 Fig). However, the F8C region of intron 2 showed a reduction of CHG and CHH methylation in *mop1-1* (Fig 1B). The F8C region was divided into two consecutive sub-regions, Int2-1 and Int2-2, to further dissect the DNA methylation affected by *mop1-1*. The 196 bp Int2-1 was previously implicated as a cob-specific regulatory region, whereas no regulatory function has been associated with the 291 bp Int2-2 region [41]. In the presence of *mop1-1*, DNA methylation in the Int2-2 region is reduced at CHG (71.9% to 41.2%)(*P* = 0.0065) and CHH (46.1% to 36.8%)(*P* = 0.0508)contexts, whereas in Int2-1 the change is not significant (Fig 1C and 1D). When the methylation level of individual cytosines was plotted for the F8C region, the reduction of CG, CHG, and CHH methylation in *P1-wr*;*mop1-1* was more pronounced in the first 78 bp of the Int2-2 region (Fig 1D). These results identified the Int2-2 region as a target of *mop1-1* mediated DNA methylation.

### Small RNA sequencing identifies regions of *P1-wr* targeted by RdDM

**Fig 2:**
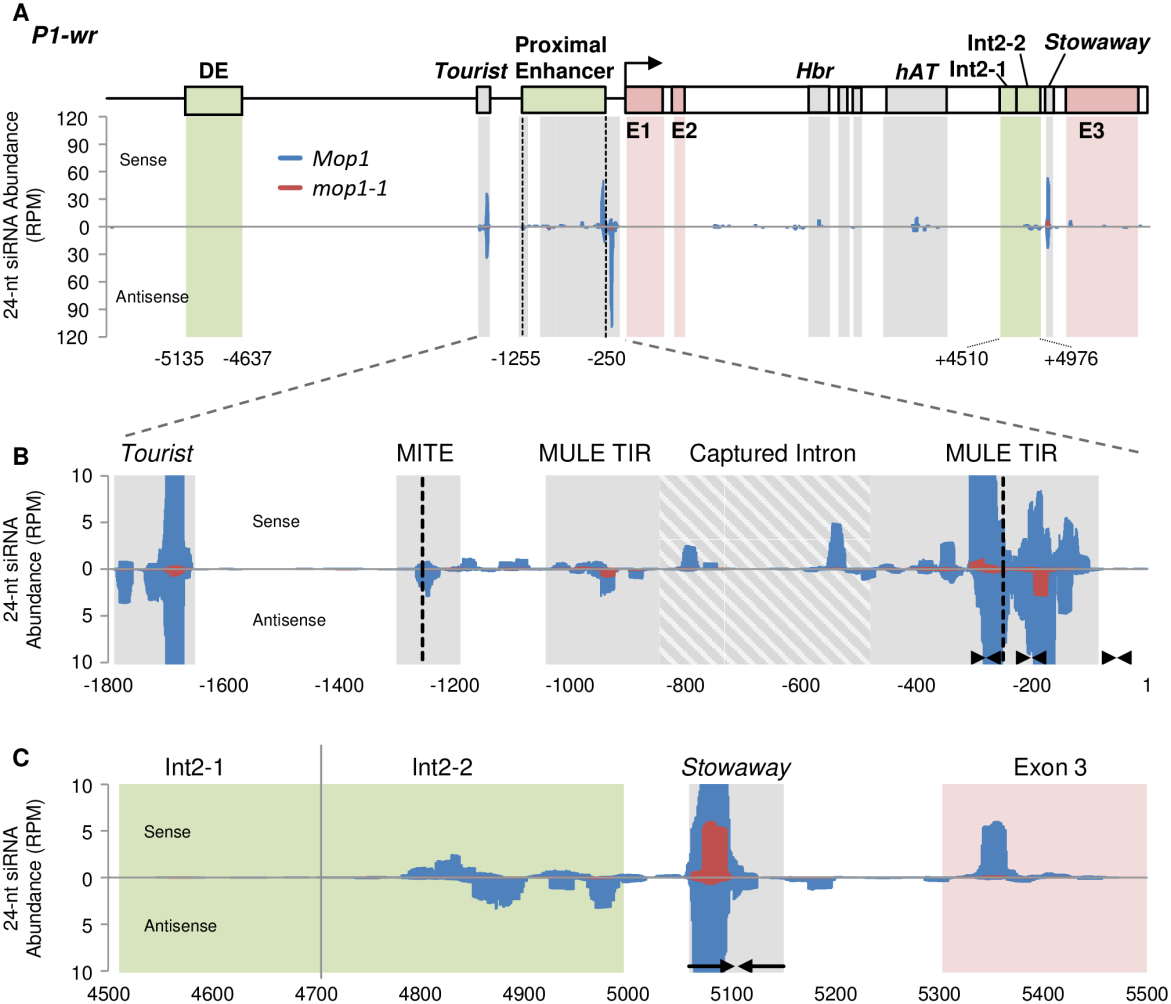
24-nt siRNA abundance at the *P1-wr* gene. (A) Line diagram of *P1-wr* showing gene structure and different regulatory elements (see Fig 1 legend for details). Abundance of 24-nt siRNA was shown on sense and antisense strands from *P1-wr*;*Mop1*/*mop1-1* (3 plants) and *P1-wr*;*mop1-1*/*mop1-1* (2 plants) samples as reads per million (RPM) and normalized to the 22-nt size class (see Methods). Green, grey, and red shaded areas indicate regulatory regions, TEs, and exons, respectively. Proximal enhancer is shown as region between black dashed lines. (B) Repetitive elements and 24-nt siRNA abundance in the proximal enhancer region. (C) 24-nt siRNA abundance in the Int2-1, Int2-2, and downstream regions. siRNA abundances from *P1-wr*;*Mop1* samples (blue) and from *P1-wr*;*mop1-1* (red) are shown. Coordinates for each graph are shown below. Black arrows indicate inverted repeats.

To identify whether the methylation of *P1-wr* was directed by siRNAs, small RNA profiling was done on young cob samples from *P1-wr*;*Mop1* and *P1-wr*;*mop1-1* plants. As expected from the function of *Mop1* and previous work [44], 24-nt siRNAs accumulation was reduced globally in *mop1-1* (S3 Fig). Small RNAs were aligned against a single copy of *P1-wr* and represented as an aggregate of sRNAs targeting this multi-copy gene. The Int2-2 region showed a complete loss of 24-nt siRNA abundance in *P1-wr*; *mop1-1* plants (Fig 2A and 2B, and Table 1), which correlates with the reduction of DNA methylation level. As shown in Fig 1D the DNA methylation in all three contexts was significantly lower in *P1-wr*; *mop1-1* plants (position 4700–4778). The lost siRNAs are downstream but adjacent to the loss of DNA methylation suggesting that the siRNAs help to recruit the machinery needed for the DNA methylation that spreads further than the siRNA targets. In addition, seven other regions of *P1-wr* had a loss of 24-nt siRNAs in *mop1-1* plants; however, these regions were not assayed for DNA methylation (See Table 1). All of the affected regions have sequences with homology to DNA transposons or are nearby such transposon sequences. One region (-325 to -155) that showed substantial reduction of siRNA accumulation contains several inverted repeat sequences of a MULE transposon (Fig 2B). This region is part of the proximal enhancer, which contains additional partial fragments of *Tourist*, MULE, and a captured intron of GRMZM2G341379. Although the distal enhancer DE has a regulatory role on *p1* expression [36], it did not show any small RNA accumulation in *Mop1* or *mop1-1*, indicating that the maintenance of DNA methylation of DE is not *Mop1*-dependent. This is consistent with the DNA methylation results for DE, which showed very little CHH methylation and the level was not affected in the absence of *Mop1* (Fig 1B). See S4 Fig for small RNA abundances for 20–23 nt classes which had very low abundance across *P1-wr*.

**Table 1:** Targets of 24-nt siRNAs accumulation at P1-wr.

Feature	Start*	End	*P1-wr; Mop1* (RPM**)	*P1-wr*; *mop1-1* (RPM)
*Tourist*	-1726	-1676	74	1
Proximal enhancer MULE	-576	-526	5	0
Proximal enhancer MULE	-376	-126	233	6
*Harbinger*	2274	2324	7	0
*hAT*	3424	3524	23	0
Int2-2	4824	4874	5	0
*Stowaway*	5024	5124	83	7
exon 3	5324	5374	7	0

*Coordinates relative to TSS, accession no. EF165349

**RPM = Reads Per Million

### *mop1-1*-induced reactivation of paramutagenic *P1-rr*’ is associated with a minor reduction of CHH methylation of the distal enhancer

**Fig 3:**
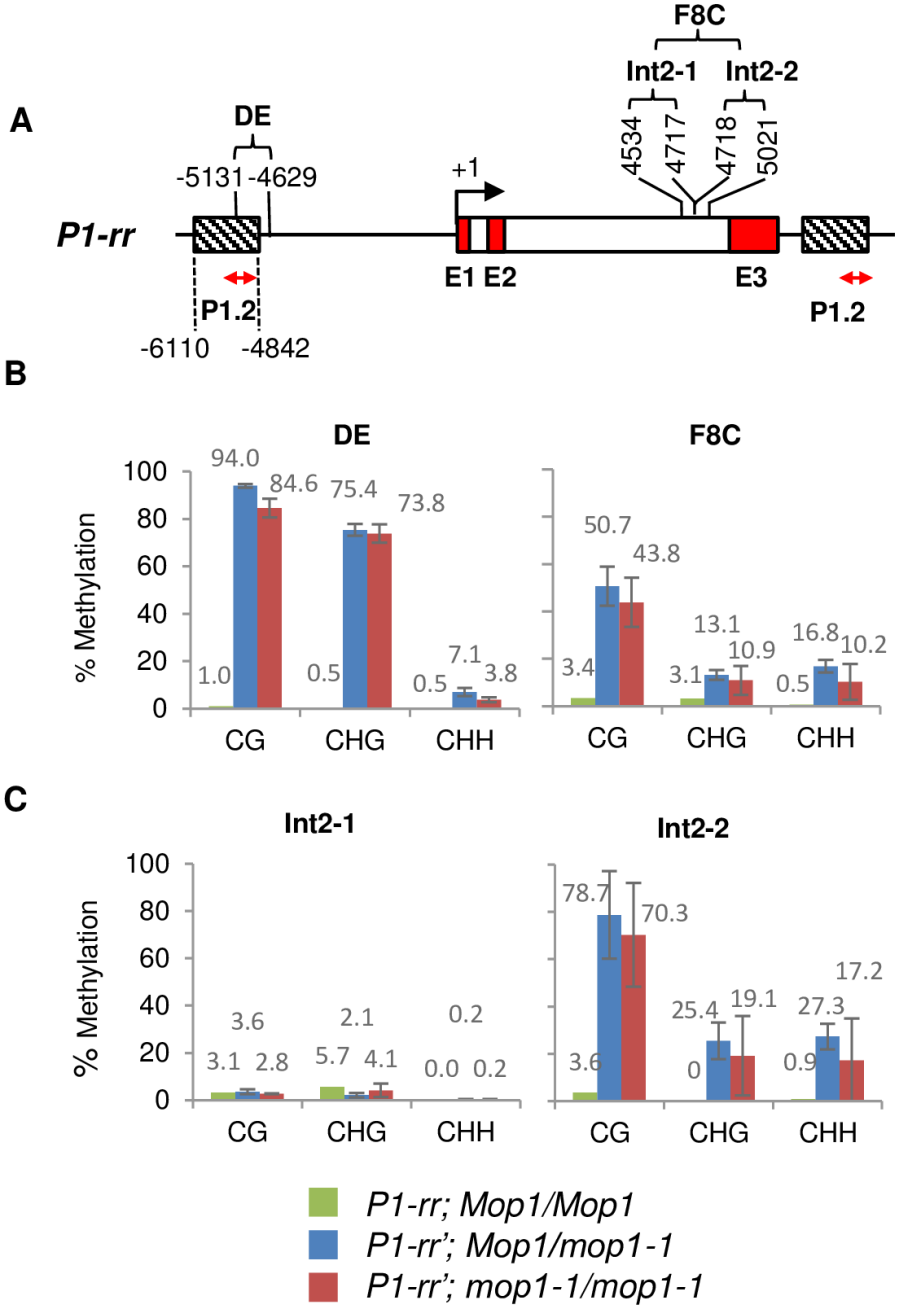
DNA methylation level of native *P1-rr* and paramutagenic *P1-rr*’ allele in Mop1 and *mop1-1* background. (A) Line diagram depicting *P1-rr* gene structure (Accession: AF427146). The exons and introns are shown as red filled and open boxes, respectively. The transcription start site is indicated by a bent arrow. The DNA methylation of the 503 bp DE region shown above the gene structure was analyzed by bisulfite sequencing. Double-headed arrow represents the region analyzed by ChIP-qPCR (Fig 5). Hatched boxes indicate the P1.2 region. (B) Average DNA methylation in CG, CHG and CHH contexts at the DE and F8C regions of *P1-rr* and *P1-rr*’ in the *Mop1* or *mop1-1* plants. (C) Average DNA methylation of the Int2-1 and Int2-2 sub-regions of F8C. The percentage methylation is shown on the y-axis. Average methylation is calculated as the mean of two biological replicates with error bars indicating the SE of the mean. Comparisons of *Mop1* and *mop1-1* plants were not significant using the Student’s *t* test at a *P* of ≤ 0.05. A single *P1-rr* sample was analyzed so there is no error bar for *P1-rr* (the data is consistent with a previous study from Sekhon et al, 2012 [39]).

**Fig 4:**
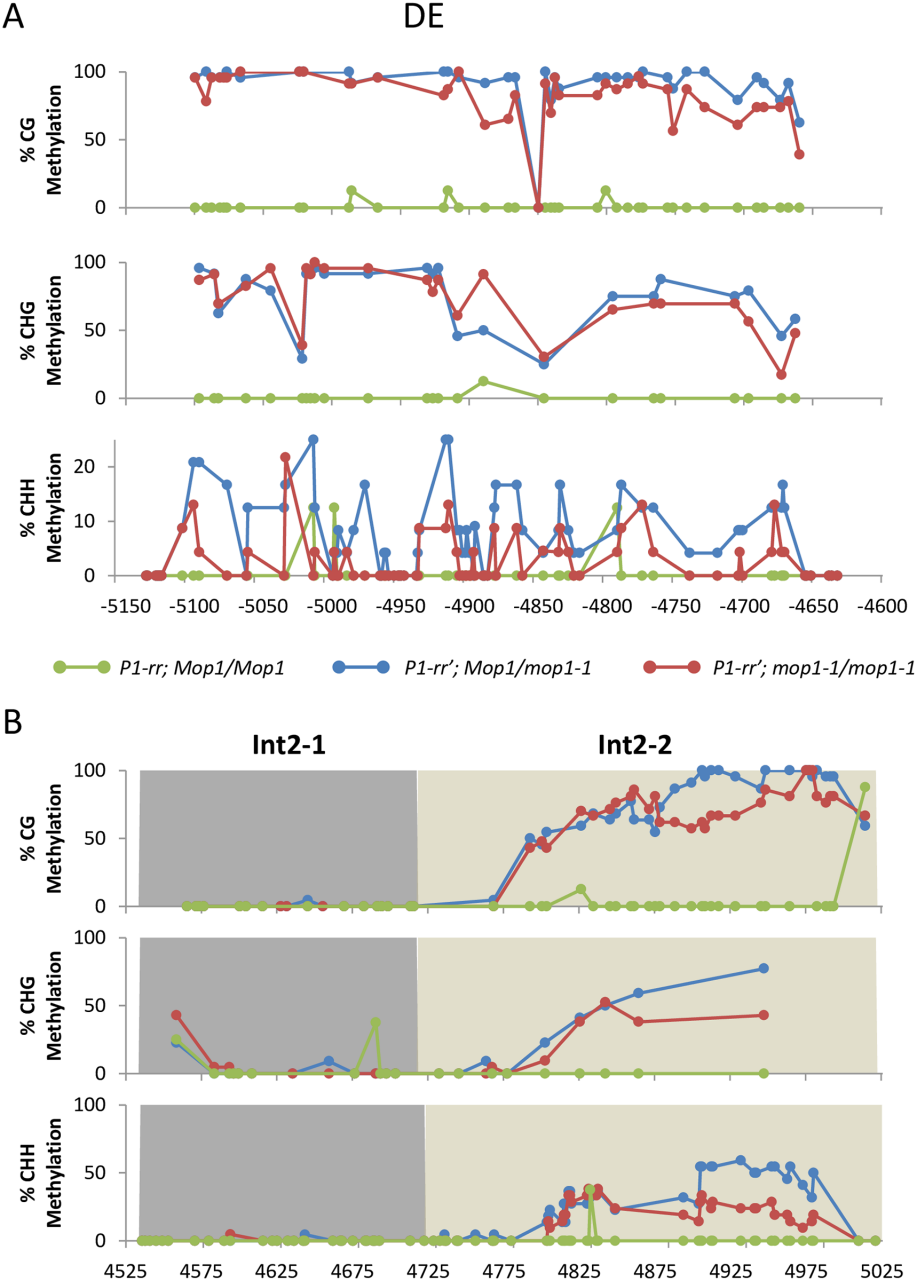
DNA methylation of individual cytosine sites of native and paramutagenic *P1-rr*alleles in *Mop1* and *mop1-1* background. Bisulfite sequencing results for individual cytosine sites in the (A) DE region and (B) Int2-1 (dark grey) and Int2-2 (light grey) regions. Each site is indicated by a circle. *P1-rr; Mop1/Mop1*, *P1-rr*’; *Mop1*/*mop1-1*, and *P1-rr*’; *mop1-1*/*mop1-1* are green, blue, and red, respectively. Data are the mean of two biological replicates (except *P1-rr* which is one plant).

To compare the regulation of a paramutagenic epiallele of *p1*, *P1-rr*’ was introgressed with *mop1-1* and *Mop1*. A previous study showed that the functional *Mop1* is required for establishing *P1-rr*’ paramutation [18]. In the *mop1-1* background, *P1-rr*’ expression was reactivated and the pericarp and cob glume pigmentation was observed. The P1.2/DE region (containing the transcriptional enhancer) is sufficient to establish transcriptional silencing associated with *p1* paramutation. Since the *P1-rr*’ silenced state is associated with hypermethylation at the DE region of *P1-rr* (Fig 3A), we investigated whether the DNA methylation would change at the *P1-rr*’ DE region in *mop1-1*. DNA bisulfite sequencing of *P1-rr* DE region showed a minor reduction in the overall symmetric and asymmetric methylation in *P1-rr*’; *mop1-1* as compared to *P1-rr*’; *Mop1* plants (Fig 3B). Notably, in the *mop1-1* homozygous plants, CG and CHH methylation were reduced from 94.0 ± 0.8% to 84.6 ± 4.0% and 7.1 ± 1.7% to 3.8 ± 1.1%, respectively. However, comparing the methylation at individual sites, most of the CHH sites which were methylated (>0%) in *Mop1* plants showed reduced methylation in *mop1-1* (Fig 4A). In addition, bisulfite sequencing of the F8C region in intron 2 revealed that Int2-1 showed low levels of DNA methylation in this region, whereas Int2-2 had statistically non-significant reductions of methylation in all contexts for *Mop1* and *mop1-1* (Figs 3C and 4B).

### *mop1-1* mediated reactivation of *P1-rr*’ is associated with reduced H3K9me2

**Fig 5:**
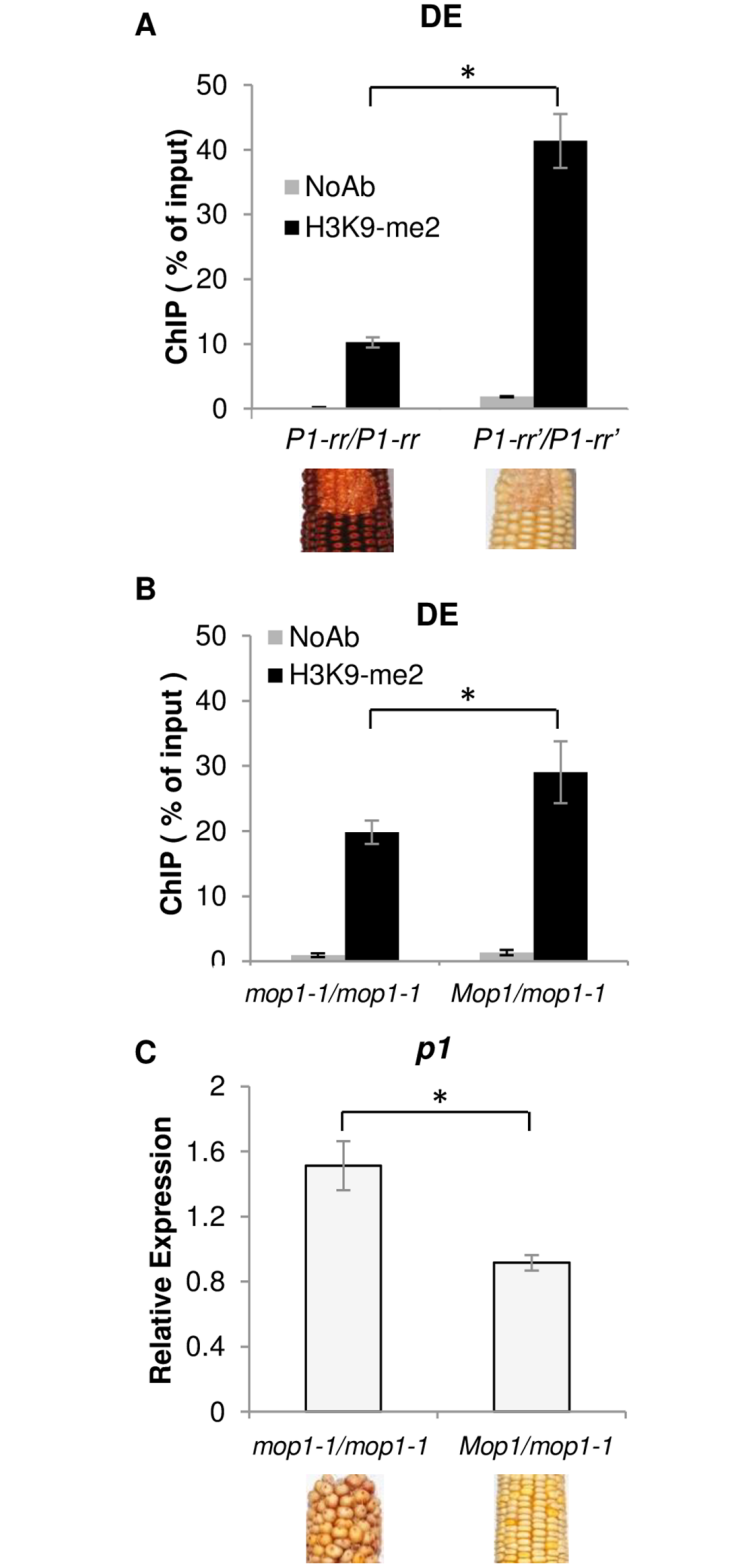
H3K9me2 and transcript analysis at *P1-rr*’ in *Mop1* and *mop1-1*. (A) ChIP-qPCR of H3K9me2 of the DE region of *P1-rr* and *P1-rr*’ plants. (B) ChIP-qPCR of H3K9me2 of the DE region of *P1-rr*’ plants in *Mop1* or *mop1-1* plants and (C) their corresponding *p1* expression from qRT-PCR. Ears are shown for the corresponding phenotypes in which the pericarp samples were used for ChIP experiments. Data are presented as the mean of three biological replicates with error bars indicating SE of the mean. * P<0.05 using an unpaired t-test.

In the absence of significant DNA methylation differences we tested if somatic silencing is associated with another chromatin modification. ChIP-qPCR was performed to compare repressive H3K9me2 mark levels with the *p1* expression using pericarps from *P1-rr*, *P1-rr*’, *P1-rr*’;*Mop1*, and *P1-rr*’;*mop1-1*. H3K9me2 was enriched three-fold within the DE in paramutagenic *P1-rr*’ compared to naïve *P1-rr* (Fig 5A). The increased methylation at both DNA and H3K9 sites in *P1-rr*’ implies that this region is under tight epigenetic suppression. The results also showed that the absence of *Mop1* caused a ~30% reduction of H3K9me2 levels within the DE region (Fig 5B). The *p1* transcripts, measured by qRT-PCR, were higher in the *mop1-1* background (Fig 5C), suggesting that reduction of H3K9me2 may be sufficient to release some level of *P1-rr*’ silencing, although several generations are required for this release.

## Discussion

The maize *p1* gene is regulated via epigenetic mechanisms that generate tissue-specific expression patterns. In addition to tissue-specific gene silencing, certain *p1* alleles/epialleles are affected by paramutation. *Mop1* is required for establishment of paramutation via biogenesis of siRNA from repetitive components of the genome. In this study two alleles of *p1* (*P1-wr* and *P1-rr*’) that differ in their paramutagenicity were used to characterize their interaction with *mop1-1*. Our results show that the tissue-specific somatic derepression of these two *p1* alleles by *mop1-1* involve different regions; this is discussed below.

### Silencing of *P1-wr* in pericarp can be attributed to two distinct regulatory regions

In a previous study, promoter::GUS transgenes of the *p1* promoter were used and two enhancers of *p1* expression were identified, the P1.2 (containing the DE region) and proximal enhancer, which may act together to give the strongest expression of *p1* [36]. In the current study, we have further characterized the key regulatory elements of *p1* that are subjects of epigenetic regulation and hence influence *p1* activity. While the DE of *P1-wr* is not a target of 24-nt siRNAs, the proximal enhancer is targeted by *Mop1*-dependent 24-nt siRNAs and likely subjected to RdDM (Fig 2). The proximal enhancer sequence contains the remnants of a MULE element with a captured intron and these transposon sequences are frequently the targets of RdDM. For example, in the case of FWA silencing, DNA methylation of the tandemly repeated promoter elements derived from ancestral transposon sequences has been well characterized [45]. Our results show these sRNAs originate from the *MULE* fragments present within this proximal enhancer of *P1-wr*. The proximal enhancer sequence similarity between of *P1-wr* and *P1-rr* alleles is over 98%. Although, we did not characterize the proximal enhancer at *P1-wr*, high levels of DNA methylation have been observed in the proximal enhancer and surrounding regions in *P1-rr* and its epiallele *P1-pr* [38]. The proximal enhancer region in these *p1* alleles resembles a *Mop1*-dependent mCHH island, regions near genes with high levels of CHH DNA methylation which mark the boundary between genic chromatin and flanking heterochromatin [46,47]. Li *et al*. (2015) identified that loss of mCHH islands in *mop1-1* can lead to reduction of CG and CHG methylation upstream of the mCHH island and can cause increased gene expression of the proximal gene. The modest up-regulation of *P1-wr* in *mop1-1* after multiple generations is possibly due to the gradual loss of repressive epigenetic marks in the proximal enhancer, although further tests of DNA methylation and/or other chromatin modifications are needed to confirm this speculation. The DE, on the other hand, may be regulated via a non-RdDM mechanism, as it is hypermethylated at symmetric sites, hypomethylated at asymmetric sites (this study, see Fig 1; Sekhon and Chopra 2009), and lacks siRNA production (this study, see Fig 2), which are some the hallmarks of non-RdDM heterochromatin [48]. This suggests that the DE is not involved in *mop1-1* activation of *P1-wr*, in contrast to its interaction with *Ufo1-1* which reduces the CG and CHG methylation of the DE [43]. This implies that *P1-wr* regulation may be attributed to a non-RdDM mechanism through the DE and a possible RdDM mediated mechanism through the proximal enhancer.

In this study, sRNA changes were also observed in another sequence, Int2-2, present within intron 2 of *P1-wr* (Fig 2, Table 1). The loss of RdDM corresponds to the reduction of DNA methylation at Int2-2 in the presence of *mop1-1* (Fig 1C and 1D). This region is nearby a *Stowaway* element from which DNA methylation may spread. This *Stowaway* element also shows loss of 24-nt sRNAs within this int2-2 region (Fig 2C). Presence of MITES in rice gene introns has been predicted to be transcribed into double stranded RNAs with a role in gene regulation [49,50]. We observed the presence of DNA methylation in the region adjoining to Int2-2 in the absence of siRNAs corresponding to that region. It has been demonstrated in maize that heterochromatin spreads from retrotransposons to the unique sequences [51]. Interestingly, there is a sharp boundary of CHH methylation between Int2-1 and Int2-2 in *Mop1* and this boundary shifts towards the *Stowaway* element in the presence of *mop1-1* (Fig 1D). Our previous study of a silenced epiallele, P1-wr*, found that the Int2-1 region conferred cob-specific *p1* gene expression [41]. *P1-wr* is a tandemly repeated multi-copy allele, so these intronic *cis* regulatory elements may act as long distance enhancers of the proximal gene copy. Combining our results, these *cis* regulatory elements in adjoining intronic sequences may thus play important tissue-specific roles in *p1* regulation.

### *P1-rr*’ silencing is maintained through H3K9me2 of the distal enhancer sequence

The P1.2 region of *P1-rr* contains *P1-wr* DE homologous sequences and has been shown to be necessary for *P1-rr*’ paramutation in addition to its role as a positive regulator of *p1* expression [13,36]. This region is repeated three times throughout the *P1-rr* gene and contains a *MULE* sequence, which makes it a likely target of RdDM. Compared to *P1-rr*, the P1.2 region of *P1-rr*’ is highly methylated and contains >7% CHH methylation, a hallmark of RdDM (Fig 3B). Indeed, the absence of *Mop1* results in about a 50% reduction of CHH methylation, but the changes of CG and CHG methylation levels are minimal (3–10% reduction see Fig 3B). In addition, our results and a previous study [39] have found that the P1.2 is also a target for epigenetic regulation via histone modifications and highly enriched for H3K9me2 in *P1-rr*’ (Fig 5A). The absence of *Mop1* leads to a reduction of the H3K9me2 inversely proportional to the up-regulation of *p1* expression (Fig 5B and 5C). A positive correlation exists between H3K9me2 levels and DNA methylation in plants as well as for several other systems [52,53]. Previous studies have shown that chromatin structure, rather than DNA methylation, is the primary effector that is responsible for silencing of an allele of the maize anthocyanin regulatory gene *Pl-Blotched* as compared with the uniformly expressed *Pl-Rhodes* allele [54]. Additionally, a chromatin-mediated mechanism was postulated when the expression of transposon *mudrA* is progressively reactivated in *mop1-1* and loss of DNA methylation precedes reactivation [27,30]. While RdDM is needed for the establishment of paramutation at *P1-rr*’, its contribution to maintenance of silencing is modest, as the silenced state of the P1.2 enhancer in *P1-rr*’ is only slightly alleviated in *mop1-1* (Figs 3–5). This indicates that after paramutation has been established, other mechanisms affecting the chromatin epigenetic state, such as histone modifications, are involved in maintaining the transcriptional silencing of *P1-rr*’. This epiallele appears to be under regulation of both RdDM and non-RdDM maintenance of its chromatin state, thus behaving like a majority of RdDM loci which only show small reductions in CG and CHG methylation in *mop1-1* [48]. It has been shown that small RNAs can be involved in maintaining high levels of H3K9me2 independent of CHG methylation in *Arabidopsis thaliana* [55]. This can explain the reduction of H3K9me2 in the *P1-rr*’ DE (Fig 5B) while CHG DNA methylation remains high in the presence of *mop1-1* (Fig 3B). Another possibility is that other chromatin-related genes down regulated in *mop1-1* [56] are causing a reduction of H3K9me2, although this seems less likely as a global reduction of H3K9me2 has not been reported for *mop1-1*. It also cannot be ruled out whether the proximal enhancer plays any role in *mop1-1* activation of *P1-rr*’; since this region is identical among *P1-wr*, *P1-rr*, and *P1-rr*’, they are likely to behave similarly.

### Sequence variation in transcriptional enhancers determine *p1* allele participation in paramutation

The P1.2 region of *P1-rr*’ is capable of transgene-induced transcriptional silencing and participates in paramutation [13,36]. It has also been demonstrated that a P1.2::GUS transgene only suppresses *P1-rr* and not *P1-wr* [13]. Our results show that *P1-rr*’ has higher CHH methylation within the DE (Fig 3B), whereas CHH sites are barely methylated at the DE of *P1-wr* (Fig 1B). In another study, a paramutagenic *P1-rr* epiallele, *P1-pr*, had slightly lower levels of CHH methylation at DE (4.5%) [35]. Because the CHH methylation is reduced by *mop1-1* it seems likely that the DE is a valid RdDM target, although at low levels. The effect of *mop1-1* at distinct regions of *P1-wr* and *P1-rr*’ may be attributed to the different small RNAs derived from structurally-modified regulatory elements of these alleles. A *MULE*, repetitive element, and a fragment of a *hAT* element are present near the DE region of the P1.2 repeat of *P1-rr*, while the DE of *P1-wr* contains only the 3’ end of the P1.2 region [37] which does not have any transposon sequences. Another allele, *p1-ww*, similarly lacks the transposon sequences in the P1.2 region and does not gain DNA methylation when heterozygous with the paramutagenic *P1-pr* [35]. A doppia element likewise has been implicated in paramutation of *r1* [57]. Similar to P1.2, the repeats that determine *b1* paramutation (*b1TR*) contain a 413 bp sequence that acts as a transcriptional enhancer and is required for transgene-induced paramutation via RNA mediated silencing [16].

### Differential requirements of somatic repression of paramutagenic alleles may be due to the balance of RdDM and maintenance methylation

The maintenance of a paramutagenic state and somatic repression in different genes has distinct requirements because these two states are variably impacted in the presence of different mutants. For example, *rmr1* is needed for somatic repression of *pl’*, but it is not needed for somatic repression of *B’* or establishment of paramutation of *pl’*, *B’*, or *R-r’* [23]. The presence of *mop1-1* immediately releases transcriptional silencing of *B’* [26]. Unlike *B’*, *P1-rr*’ silencing is not immediately released by *mop1-1* [18]. The only other RdDM mutant that has been tested with *P1-rr*’ is *Mop2-1*, which is needed for establishment of paramutation but not for somatic repression [21]. The uncloned *Ufo1-1* mutant has also been shown to be required for somatic repression of *P1-rr*’ and *B’*, however its role in the establishment of paramutation at either loci has not been reported [39]. Our study has demonstrated that symmetric DNA methylation of the important paramutagenic region of *p1* remains high in the presence of *mop1-1* even after multiple generations of exposure. Thus the DE of *P1-rr*’ appears to be the target of *Mop1*-mediated RdDM for maintenance of paramutagenicity and *Mop1*-independent maintenance of CG and CHG methylation, needed to maintain somatic repression. This could explain the different responses of *B’* and *P1-rr*’ to *mop1-1*, with *B’* somatic repression depending mainly upon RdDM. One possibility for this difference in behavior is that the DE is proximal to transposon sequences which are subject to heterochromatic silencing whereas the *b1TRs* do not contain such sequences [15]. Future studies of *P1-rr*’ with additional mutants would further elucidate the complexity of chromatin regulation needed for various aspects of paramutation in maize.

## Conclusions

Alleles of the maize *p1* gene display unique and tissue-specific expression patterns and contain regions important for transcriptional regulation. This study identifies the proximal enhancer as a candidate for *Mop1*-mediated somatic repression of *P1-wr*, while also showing *Mop1* does not affect the known pericarp distal enhancer element. We also provided evidence for the basis of the differential requirement of *Mop1* for somatic silencing of *P1-rr*’ as compared to *B’*. Namely, the sequences necessary for paramutation have overlapping silencing mechanisms, such that even in the presence of *mop1-1*, symmetric DNA methylation and H3K9me2 remain relatively high compared to the fully-expressed, paramutable allele. Similar mechanisms might be present at other paramutagenic loci which have different requirements for establishing paramutation and maintaining somatic repression. This also raises the question of why *P1-rr*’ does not fully transition into non-RdDM maintenance of silencing a question which may be addressed by further study of the non-paramutagenic epiallele *P1-pr*^*tp*^ as well as additional RdDM mutants.

## Materials and methods

### Genetic stocks, genetic crosses and genotyping

All of the populations discussed here were planted at the Penn State University Agronomy Farm, Rock Springs, PA.

In the *P1-wr*; *mop1-1* study, the *P1-wr* allele has been exposed for three generations to *mop1-1* mutant background and the detailed crossing scheme has been described in details previously (Sidorenko and Chandler, 2008). Briefly, the *P1-wr* stock carrying *Mop1*/*mop1-1* was outcrossed to a *mop1-1*/*mop1-1* plant. The subsequent generation was self-pollinated and the plants carrying *P1-wr/P1-wr*; *mop1-1*/*mop1-1* were designated as mutants and selected using the similar method as explained below for *P1-rr*’ allele. Plants with genotype *P1-wr/P1-wr*; *Mop1*/*mop1-1* genotype were used as wild types.

The paramutagenic *P1-rr*’ allele was derived from a transgene-induced silencing event from a cross between *P1-rr* and a transgenic line containing a P1.2b:: GUS transgene (Sidorenko and Peterson, 2001). The *P1-rr*’ stock used in this study was the progeny of a homozygous (*P1-rr’/P1-rr’*) plant that showed very strong silencing and had colorless pericarps and light pink to colorless cob glumes (Sidorenko and Peterson, 2001). The progenies were generated by intercrossing *P1-rr’/P1-rr’*; *Mop1*/*mop1-1*; *B*’ and *P1-rr’/P1-rr’*; *mop1-1*/*mop1-1*; *B’* (S2 Fig). These progeny plants were further genotyped by multiplex PCR using specific primers to distinguish homozygous and heterozygous *mop1-1* plants. The primers used for genotyping are listed in S1 Table and PCR conditions are as follows: 94°C for 5 min, 30 cycles of (94°C for 30 sec, 56°C for 45 sec, 72°C for 45 sec), and final extension step at 72°C for 10 min. Increased plant pigmentation (purple plant) conferred by up-regulation of the *B’* was used to identify the *mop1-1* mutant (*mop1-1*/*mop1-1*) plants. The progenies showing gain of pericarp pigmentation used for molecular analysis have been exposed to *mop1-1* background for three generations. Plants carrying homozygous and heterozygous *mop1-1* were further selected as mutants and wild types respectively, for molecular characterization including DNA methylation, chromatin modifications, and gene expression analysis. The control *P1-rr* allele used in this study was derived from the standard *P1-rr-4B2* genetic stock (Grotewold et al., 1991).

### Plant genomic DNA extraction and genomic bisulfite sequencing

Leaf (v6 stage) and pericarp (18 days after pollination; DAP) tissue samples were harvested, snap frozen in liquid nitrogen, and stored at -80°C. Leaf genomic DNA was used from *P1-wr*/*P1-wr*; *Mop1*/*mop1-1* and *P1-wr*/*P1-wr*; *mop1-1*/*mop1-1*) plants. Pericarp tissues (18 DAP) from *P1-rr*’ carrying homozygous (red) and heterozygous (colorless) *mop1-1* plants were used for bisulfite sequencing assays. Genomic DNA was extracted using a modified CTAB (cetyltrimethylammonium bromide) method [58]. DNA was precipitated with 7.5M ammonium acetate and the pellet was re-suspended in 1X TE (1 M Tris pH 8.0 and 0.5 M EDTA pH 8.0). The genomic DNA from two independent plants was used for the bisulfite sequencing analysis. Bisulfite treatment was performed with EZ DNA Methylation-Gold Kit (Zymo Research, Orange, CA) and PCR amplified using gene-specific primers (S1 Table). To get high-yielding PCR products from bisulfite treated DNA, nested PCR was performed using two pairs of primers. For the distal enhancer region (fragment 15), RBS8F and RBS1R were used as external primers, and then RBS9F and RBS3R were used as nested primers to amplify a 499-bp region. For *P1-rr*, the DE region is 503 bp. For the 3’ end of intron 2 (fragment 8C), RBS11F and RBS11R were used as external primers, and then RBS12F and RBS12R were used as nested primers to amplify a 487-bp region. All PCR products were subcloned into pSC-A-amp/kanamycin vector using StrataClone PCR Cloning Kit (Agilent Technologies, Santa Clara, CA). The plasmid DNA was isolated using a StrataPrep Plasmid Miniprep Kit (Agilent Technologies, Santa Clara, CA) and DNA sequencing was performed using ABI Hitachi 3730XL DNA Analyzer in the Penn State Nucleic Acid Facility. For each bisulfite sequencing experiment, two biological replicates were used and at least ten technical replicates (clones) were obtained to determine the average methylation per sample. The DNA sequence data was aligned using ClustalW (http://www.ebi.ac.uk/Tools/msa/clustalw2/). To analyze the DNA methylation patterns of the compiled clones for each plant from each genotype, CyMATE was used to illustrate each DNA methylation sequence context (CG, CHG, CHH) (Yu et al., 2008). Overall DNA methylation in each context was calculated by dividing the number of methylated cytosines in a given context by total number of cytosines in that context in all clones. Average methylation is calculated as the mean of biological replicates with error bars indicating SE of the mean. The percent methylation between genotypes was compared with Student’s *t* tests and were considered significant at a *P* ≤ 0.05.

### Small RNA sequencing and analysis

Young cobs (4 cm) were collected from three wild type (*Mop1*/*mop1-1*) and from two *mop1-1* plants from a BC1F2 population segregating for *mop1-1*. For small RNA libraries, total RNA from the materials described above was isolated using Tri Reagent^™^ (Molecular Research Center, Inc., Cincinnati, OH). Small RNA libraries were constructed using the Illumina TruSeq Small RNA Sample Preparation Kit (RS-200-0012), and sequenced on an Illumina HiSeq2000 instrument at University of Delaware. Raw sequencing data was first trimmed of adapter sequences and then the read counts were normalized based on the total abundance of genome-matched reads, excluding structural sRNAs originating from annotated tRNA, rRNA, small nuclear (sn) and small nucleolar (sno) RNAs. The genome sequences were from maize B73 RefGen_v2. The small RNA sequence data are available from NCBI's Gene Expression Omnibus (GEO) under GEO Series accession number GSE68510 https://www.ncbi.nlm.nih.gov/geo/query/acc.cgi?acc=GSE68510. Small RNA reads were aligned to the *P1-wr* gene (GenBank: EF165349) using bowtie. Only reads with perfect match to *p1* sequences were kept. Read counts were normalized to 1M reads per library as well as to 22 nt abundances. The 22-nt abundances were used for normalization control as *mop1-1* causes a drastic reduction of 24-nt siRNA that leads to an overrepresentation of other size classes [44]. A single copy of the *P1-wr* sequence was analyzed as a representative for the remaining copies. Read depth across the gene was calculated using Samtools. To assess regions targeted by 24-nt siRNAs, reads were counted in 50 bp bins across the gene and results shown in Table 1.

### Reverse transcription and Quantitative Real-Time PCR

Total RNA was isolated from pericarp tissues using RNAzol following the standard protocol (Molecular research center, Cincinnati, OH). The first strand cDNA templates were reverse transcribed with Superscript III reverse transcriptase (Invitrogen, Grand Island, NY) using oligo dT as a primer. Quantitative real-time PCR (qRT-PCR) was performed with ABI7500 Fast real-time PCR system using SYBR Green I (Roche, Madison, WI) as the detection system and the default program: 10 minutes of pre-incubation at 95°C followed by 40 cycles of (95°C for 15 sec, 60°C) for 1 min for PCR amplification. The primers used for qRT-PCR are *p1* primers (RT_PWREx_2F and RT_PWREx_2R) and *actin* primers (actin_exon2_Fw and actin_exon2_Rev) (S1 Table). *Actin1* gene was used as an endogenous control. The relative expression level of each gene was calculated using the 2^–ΔΔCt^ method (Livak and Schmittgen, 2001). Gene expression analysis was performed with three independent samples.

### Chromatin immuno-precipitation assay and quantitative real-time PCR (ChIP-qPCR)

ChIP assays were performed using pericarp tissues following a modified protocol (Haring et al., 2007; Kimura et al., 2008). Briefly, 18-day-old pericarp tissues were cross-linked with 3% formaldehyde and the chromatin complex was extracted and sheared to a size range of 0.5 to 1 kb fragments using a Bioruptor (Diagenode, Denville, NJ). The antibody used for ChIP was an anti-H3K9me2 kindly provided by Dr. Hiroshi Kimura [59]. This antibody was then coupled with sheep anti-mouse IgG Dynabeads M-280 (Life technologies, Carlsbad, CA). In addition, normal mouse IgG was used as a no-antibody control (NoAb). The ChIPed DNA was further purified using QIAquick PCR Purification Kit (QIAGEN, Valencia, CA) and quantified with qPCR. The primers used here were specific to distal enhancer (DE) region (PW_RTF15-2_Fw and PW_RTF15-2_Rev) as listed in S1 Table. ChIP-qPCR data was normalized using percentage-of-input method (See the details of ChIP analysis online guide: https://www.thermofisher.com/us/en/home/life-science/epigenetics-noncoding-rna-research/chromatin-remodeling/chromatin-immunoprecipitation-chip/chip-analysis.html). The relative differences between ChIP assays and input samples were determined using the Δ*C*_*T*_ method and presented as a percentage of the input (taken as 100%). Data shown in this study are representative results of three independent experiments.

## Supporting information

S1 Fig Cytosine DNA methylation level of of 499-bp *P1-wr* distal enhancer (DE) region in *mop1-1* and *Mop1* plants. Methylation profile at CG, CHG and CHH contexts were obtained by genomic bisulfite sequencing. The y-axis shows percentage of DNA methylation. Individual sites are shown as circles.
(TIF)

S2 Fig Crossing scheme to generate a segregating population from *P1-rr*’/*P1-rr*’; *Mop1*/*mop1-1* and *P1-rr*’/*P1-rr*’;*mop1-1*/*mop1-1*. Crosses between *P1-rr’/P1-rr’* carrying homozygous (*mop1-1*/*mop1-1*) and heterozygous (*Mop1*/ *mop1-1*) were made to test whether *mop1-1*-induced reactivation of *P1-rr*’ is associated with hypomethylation. Since the stocks also carried a silenced *B’* allele, plants containing homozygous *mop1-1* were identified by the purple color of plant body, whereas plants containing heterozygous *Mop1*/*mop1-1* showed green plant without purple pigmentation.
(TIF)

S3 Fig Size distributions of young ear small RNAs. (A) Size distribution of small RNAs in *P1-wr*;*Mop1* and *P1-wr*;*mop1-1* samples with sum of small RNA abundances normalized to 1 million reads. (B) Size distribution of small RNA abundance after normalization to the abundance of 22-nt class. Three independent *Mop1* samples and two independent *mop1-1* samples are shown.
(TIF)

S4 Fig siRNA abundance of different size classes at the *P1-wr* gene. (A) Line diagram of *P1-wr* showing gene structure and different regulatory elements (see Fig 1 legend for details). (B) Abundance of siRNA was shown on sense and antisense strands from *P1-wr*;*Mop1*/*mop1-1* and *P1-wr*;*mop1-1*/*mop1-1* samples as reads per million (RPM) and normalized to the 22-nt size class (see Methods). Green, grey, and red shaded areas indicate regulatory regions, TEs, and exons, respectively. Proximal enhancer is shown as region between black dashed lines. siRNA abundances from *P1-wr*;*Mop1*/*mop1-1* samples (blue) and from *P1-wr*;*mop1-1*/*mop1-1* (red) are shown.
(TIF)

S1 Table Oligo sequences. (XLSX)
