# Supplementary figures and images for: Overlapping RdDM and non-RdDM mechanisms work together to maintain somatic repression of a paramutagenic epiallele of maize pericarp color1
Source: PLoS One. 2017 Nov 7;12(11):e0187157. doi: 10.1371/journal.pone.0187157 (PMC5675401; doi:10.1371/journal.pone.0187157)

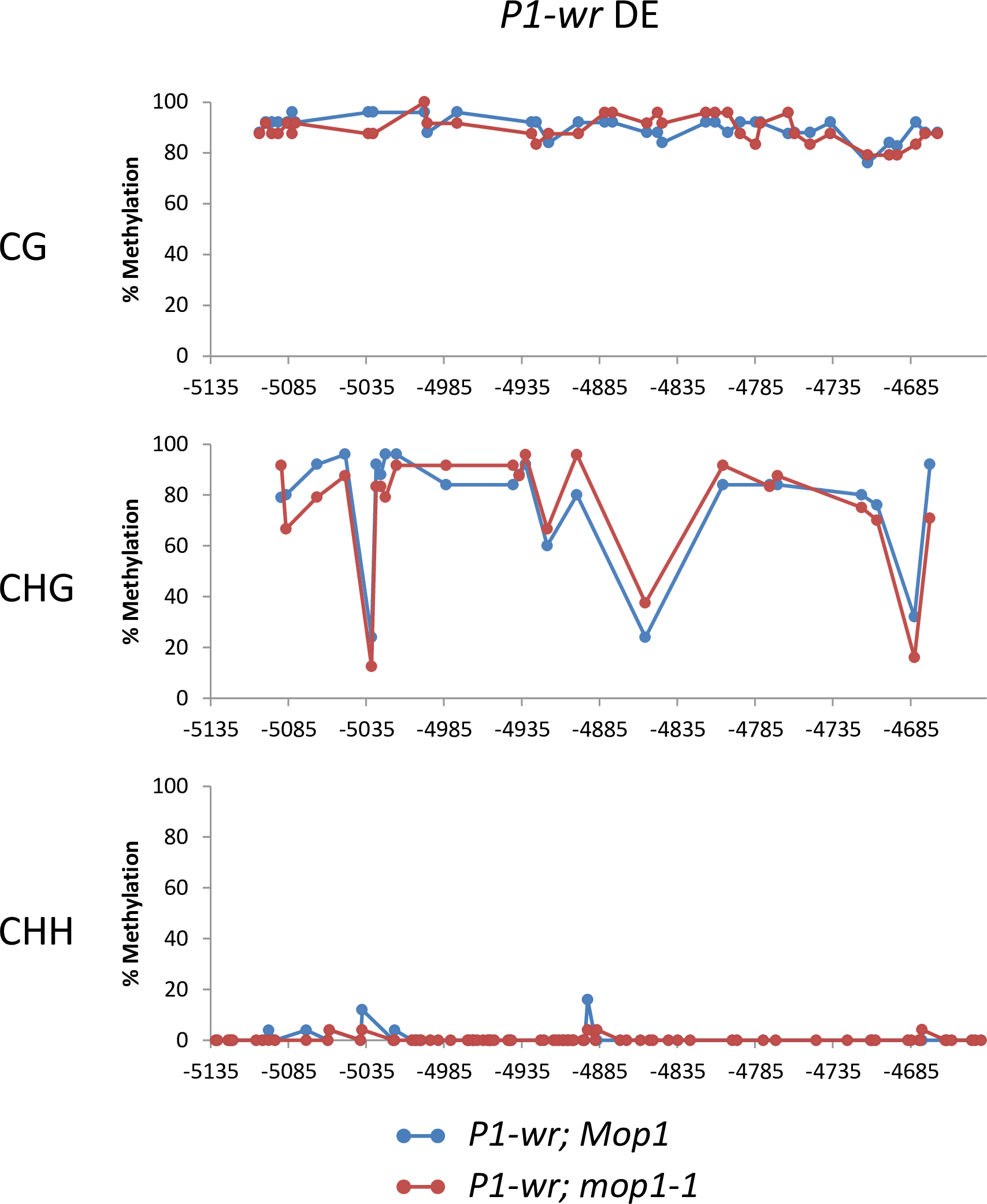

Supplement: S1 Fig — Methylation profile at CG, CHG and CHH contexts were obtained by genomic bisulfite sequencing. The y-axis shows percentage of DNA methylation. Individual sites are shown as circles. (TIF) [file pone.0187157.s001.tif]

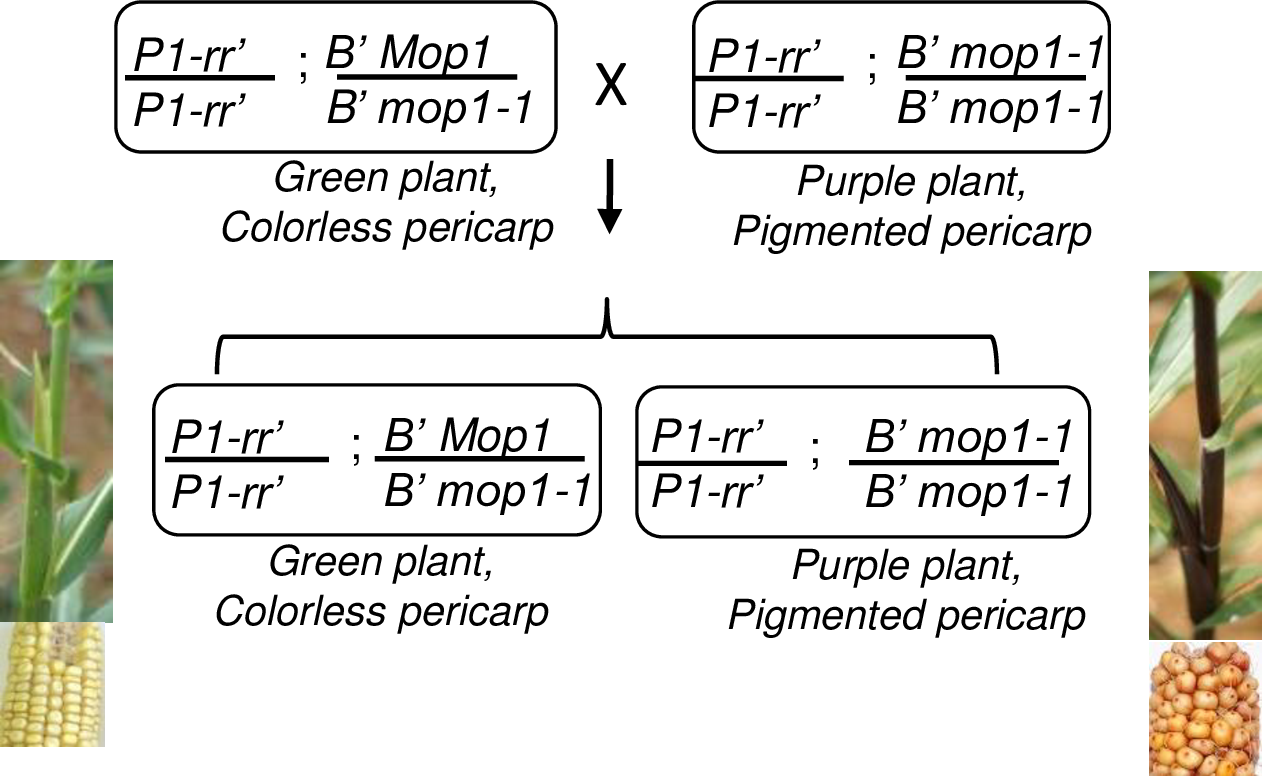

Supplement: S2 Fig — Crosses between P1-rr’/P1-rr’ carrying homozygous (mop1-1/mop1-1) and heterozygous (Mop1/ mop1-1) were made to test whether mop1-1-induced reactivation of P1-rr’ is associated with hypomethylation. Since the stocks also carried a silenced B’ allele, plants containing homozygous mop1-1 were identified by the purple color of plant body, whereas plants containing heterozygous Mop1/mop1-1 showed green plant without purple pigmentation. (TIF) [file pone.0187157.s002.tif]

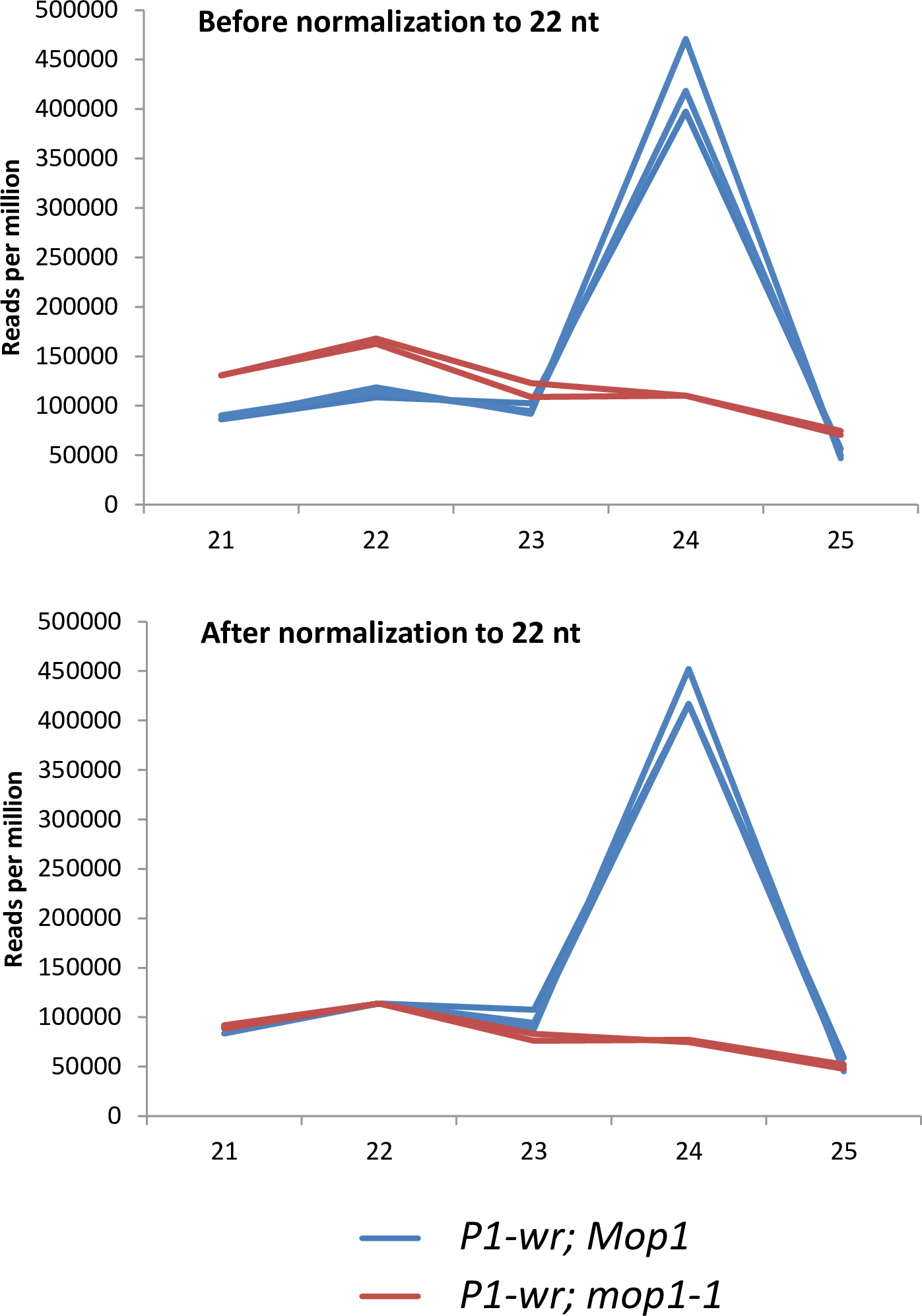

Supplement: S3 Fig — (A) Size distribution of small RNAs in P1-wr;Mop1 and P1-wr;mop1-1 samples with sum of small RNA abundances normalized to 1 million reads. (B) Size distribution of small RNA abundance after normalization to the abundance of 22-nt class. Three independent Mop1 samples and two independent mop1-1 samples are shown. (TIF) [file pone.0187157.s003.tif]

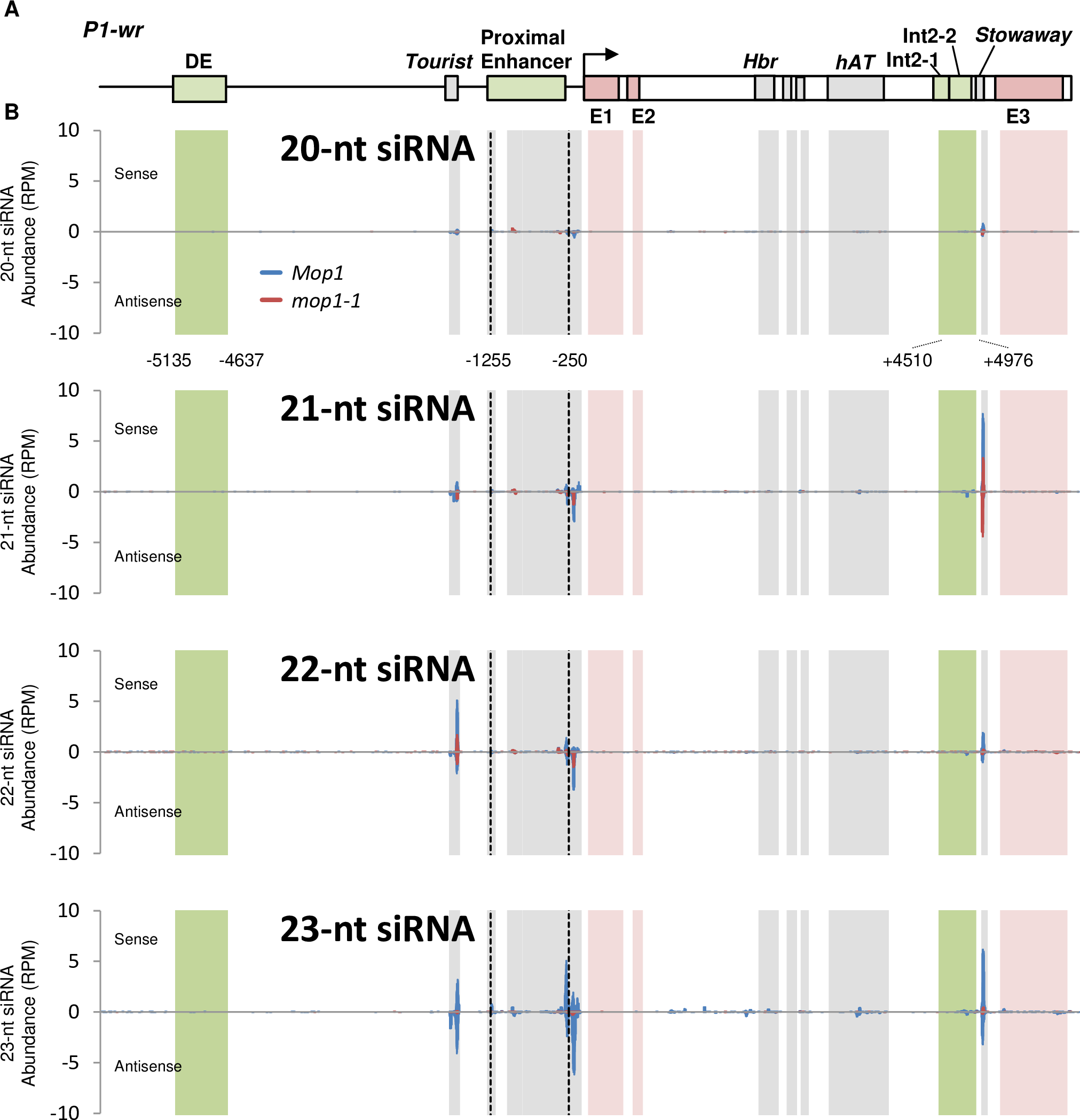

Supplement: S4 Fig — (A) Line diagram of P1-wr showing gene structure and different regulatory elements (see Fig 1 legend for details). (B) Abundance of siRNA was shown on sense and antisense strands from P1-wr;Mop1/mop1-1 and P1-wr;mop1-1/mop1-1 samples as reads per million (RPM) and normalized to the 22-nt size class (see Methods). Green, grey, and red shaded areas indicate regulatory regions, TEs, and exons, respectively. Proximal enhancer is shown as region between black dashed lines. siRNA abundances from P1-wr;Mop1/mop1-1 samples (blue) and from P1-wr;mop1-1/mop1-1 (red) are shown. (TIF) [file pone.0187157.s004.tif]
